# Transgenerational adaptation to hypoxia

**DOI:** 10.1126/sciadv.adv9451

**Published:** 2025-10-24

**Authors:** Kathleen Kim, Ariel Telger, Gautam Sarkar, Sharan Surya, M. Hafiz Rothi, Michael P. Meers, Simon Yuan Wang, Eric Lieberman Greer

**Affiliations:** ^1^ Department of Pediatrics, HMS Initiative for RNA Medicine, Harvard Medical School, Boston, MA 02115, USA.; ^2^ Division of Newborn Medicine, Boston Children’s Hospital, Boston, MA 02115, USA.; ^3^ Department of Pediatrics, Washington University School of Medicine, St. Louis, MO 63110, USA.; ^4^ Department of Developmental Biology, Washington University School of Medicine, St. Louis, MO 63110, USA.; ^5^ Department of Molecular Biology, Massachusetts General Hospital, Boston, MA 02114, USA.; ^6^ Department of Genetics, Washington University School of Medicine, St. Louis, MO 63110, USA.

## Abstract

Epigenetic inheritance alerts naïve descendants to prepare for stresses that could still be present, whereas distant descendants return to a basal state after several generations without stress. However, organisms are frequently exposed to stresses successively across generations. We found that parental hypoxia exposure increased P0 longevity, caused intergenerational lipid reduction, and elicited transgenerational fertility reduction that was dependent on generationally transmitted small RNAs. Here, we find that *Caenorhabditis elegans* adapt to repeated generational stresses. We show that, upon two repeated generational hypoxia exposures, the life-span extension is eliminated, and after four repeated generational hypoxia exposures, the reduced fertility is eliminated. Transgenerational adaptation also occurred in response to changes in glucose availability. Transgenerational hypoxia adaptation is dependent on the H3K27 trimethyltransferase PRC2 complex, and we identified transgenerationally adapted genes. Our findings reveal that transgenerational adaptation occurs and suggest that H3K27me3 is a critical modification for adapting to repeated generational stresses.

## Teaser

Demonstration of adaptation on a multigenerational scale to repeated stresses.

## INTRODUCTION

Most genetic information is encoded and inherited through DNA (*1*). However, previous findings demonstrated that changes in gene expression can be mediated in the absence of changes to the DNA sequence. Heritable phenotypes regulated by epigenetic information include physical appearance (*2*, *3*), metabolism (*4*), behavioral state (*5*), and longevity (*6*, *7*). The phenomena of heritable nongenetic information being transmitted across generations presumably evolved to allow organisms to pass important information to their descendants, allowing their progeny to better survive altered environments without mutations to their genome. By avoiding mutation, the organism can survive irregular conditions but returns to a basal state when the environmental conditions improve (*8*). However, how does the adaptable nature of epigenetics (*9*) respond to repeated environmental stresses? Can repeated environmental stresses override the canonical epigenetic inheritance? Is there adaptation on a transgenerational scale as well?

Although laboratory experiments have mostly focused on conditions of a transient environmental stress followed by a return to basal environmental conditions, nature rarely follows such controlled conditions. Organisms are frequently exposed to stresses repeatedly over their lifetime or over a generational timescale or alternatively exposed to one stress in one generation and then a second stress in a subsequent generation. Here, we asked whether repeated generational exposure to stresses altered the generational inheritance clock, overrides canonical epigenetic inheritance, or changed the magnitude of the phenotypic response.

Low oxygen (hypoxia) is an environmental stress, which can be harmful in many contexts such as inducing ischemia and eventually cell death (*10*). The hypoxic tumor microenvironment is also correlated with increased metastasis and mortality (*11*). Fetal hypoxia impairs development and is associated with several pregnancy disorders and complications (*12*–*15*). Ocean warming and acidification caused by climate change have lowered ocean oxygen levels and therefore are threatening marine life (*16*–*18*). In mammalian cells, in response to hypoxia, the H3K27me3 demethylase KDM6A/UTX senses lowered oxygen levels, causing elevated H3K27me3 levels and blocks cellular differentiation (*19*). *Caenorhabditis elegans* frequently encounter oxygen depletion in nature when the soil gets flooded (*20*). We and others have previously found that hypoxia increases *C. elegans* life span, reduces neutral lipids, and reduces fertility in the exposed generation (P0) (*21*–*24*). We previously found that hypoxia exposure in the P0 generation causes an intergenerational (F1) reduction in neutral lipids and a transgenerational (F1+F2) reduction in fertility accompanied by a dysregulation of gene expression (*24*). We found that the transgenerational epigenetic inheritance of hypoxia-induced fertility defects was dependent on the small RNA argonaute HRDE-1, the putative H3K9 trimethyltransferase SET-25, the putative H3K27 trimethyltransferase MES-2, and the putative H3K27me3 demethylase JMJD-3.2 (*24*). We further found that small RNAs isolated from hypoxia-treated *C. elegans* are sufficient to elicit fertility defects and identified transgenerationally dysregulated small RNAs. We identified that one specific double-stranded RNA (dsRNA) against *F44E5.4/5* is sufficient to induce a heritable decrease in fertility and is directly transmitted from parents to their progeny (*24*). However, it is still unclear whether animals will adapt to repeated generational exposure to hypoxia.

Here, we show that *C. elegans* that were exposed to hypoxia repeatedly over several generations failed to display hypoxia-induced phenotypes. We found that upon two repeated generational exposures to hypoxia, *C. elegans* no longer displays an increase in life span, and after four repeated generational exposures to hypoxia, *C. elegans* no longer displays a decrease in reproduction. In addition, this transgenerational adaptation was not unique to hypoxia—exposure to 1% glucose caused a subtle increase in neutral lipids and a decrease in fertility, whereas two repeated generational exposures to elevated glucose eliminated the capacity of *C. elegans* to respond to the elevated glucose to alter lipid content; after three repeated generational exposures to glucose, *C. elegans* no longer displays a decrease in reproduction. The transgenerational adaptation of decreased reproduction to hypoxia is dependent on the putative H3K27 trimethytransferase PRC2 complex, and we identified critical genes that adapted on a transgenerational time frame to repeated hypoxia exposure. Together, our results reveal that transgenerational adaptation occurs and suggest that H3K27me3 is a critical modification for adapting transgenerationally to repeated generational stresses.

## RESULTS

### *C. elegans* adapt to repeated hypoxia exposure

**Fig. 1.**
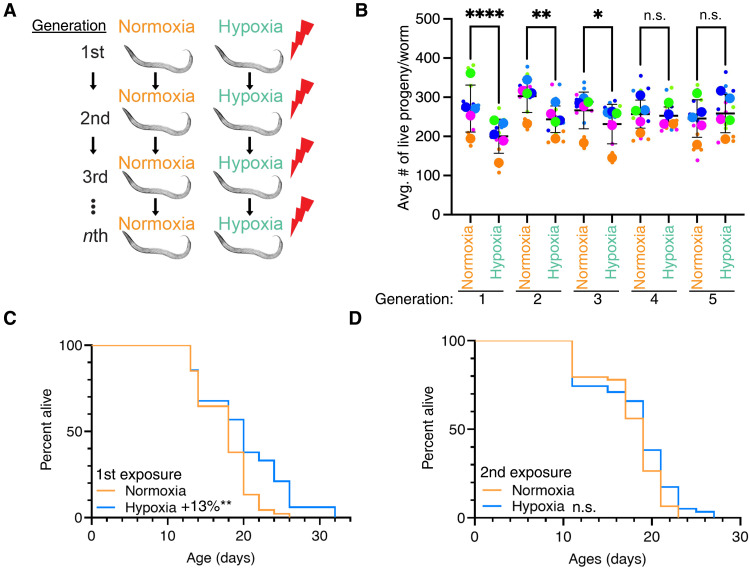
*C. elegans* adapt transgenerationally to repeated hypoxia exposure. (**A**) Scheme of the repeated generational hypoxia exposure. (**B**) Repeated generational hypoxia exposure leads to decreased fertility for three successive generations but fails to induce a significant fertility defect after four repeated exposures. Each color-coded dot represents an individual experiment performed in triplicate with 10 worms per plate, and each column represents the means ± SEM. *P < 0.05; **P < 0.01; ****P < 0.0001; not significant (n.s.), *P* > 0.05, as assessed by Fisher’s combined probability test. Data are presented in superplot format (*65*) with the average of each experiment shown in a larger colored dot. (**C** and **D**) Hypoxia exposure induces life-span extension after one exposure (C) but not after two generational exposures (D). Statistics are presented in table S1.

Hypoxia exposure increases life span and decreases fertility (*24*, *25*). To determine whether *C. elegans* can transgenerationally adapt to stresses, we repeatedly exposed *C. elegans* to hypoxia across multiple generations (Fig. 1A) and measured reproduction and longevity. We exposed larval stage (L4) worms to hypoxia (0.1% oxygen) for 16 hours before returning to a normoxic environment (~21% oxygen). We subsequently exposed F1, F2, F3, and F4 descendants to similar hypoxia periods (Fig. 1A) and measured progeny production at each generation. We found that the P0, F1, and F2 generations all displayed reduced fertility in response to hypoxia treatments; however, by the fourth repeated generational exposure to hypoxia, *C. elegans* no longer displayed a hypoxia-induced fertility decrease (Fig. 1B and table S1). This adaptation persisted as the fifth repeated generational exposure to hypoxia also no longer displayed a hypoxia-induced fertility decrease. We found that the generational adaptation to hypoxia occurred more rapidly for longevity. Whereas the initial exposure to hypoxia subtly increased *C. elegans* life span (Fig. 1C and table S2; 13% P = 0.0022 by combined Fisher’s test), as has been shown previously (*24*, *25*), *C. elegans* exposed to hypoxia, whose parents had also been exposed to hypoxia, did not display an increase in life span (Fig. 1D and table S2; P = 0.1495 by combined Fisher’s test). Together, these findings suggests that *C. elegans* transgenerationally adapt to become resistant to hypoxia conditions after repeated generational exposure.

### *C. elegans* adapt to repeated changes in glucose availability

**Fig. 2.**
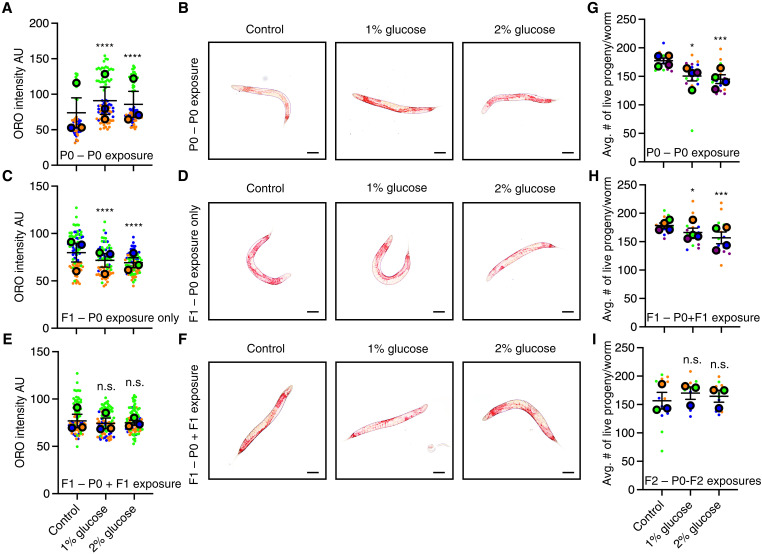
*C. elegans* adapt transgenerationally to repeated elevated glucose exposure. (**A** and **B**) Exposure to 1 or 2% glucose leads to elevated neutral lipid levels as assessed by oil red O (ORO) staining in the parental P0 generation. (A) Each large dot represents replicate experiments performed with 13 to 34 worms. AU, arbitrary units. (B) Representative images of the P0 exposed generation. (**C** and **D**) Exposure to 1 or 2% glucose in parental generation leads to decreased levels of neutral lipids in the F1 unexposed progeny by ORO staining. (C) Each large dot represents replicate experiments performed with 13 to 38 worms. (D) Representative images of the F1 progeny. (**E** and **F**) Repeated exposure to 1 or 2% glucose in the parental and F1 generation eliminates the increase in neutral lipids observed in the P0 generation as assessed by ORO staining. (E) Each large dot represents replicate experiments performed with 18 to 55 worms. (F) Representative images of the F1 progeny exposed to different amounts of glucose, whose parents were also exposed to those altered glucose levels. (**G**) Exposure to 1 or 2% glucose leads to decreased fertility. Each large dot represents an individual experiment performed in triplicate with 10 worms per plate. (**H**) Two successive generational exposures to 1 or 2% glucose lead to decreased fertility. (**I**) Three successive generational exposures to 1 or 2% glucose fail to induce decreased fertility. In all graphs, each color-coded dot represents the means ± SEM with individual replicates within an experiment presented in the same color with a smaller dot in superplot format (*63*). *P < 0.05; ***P < 0.001; ****P < 0.0001; ns, *P* > 0.05, as assessed by Fisher’s combined probability test.

To determine whether transgenerational adaptation is specific to hypoxia or is a general phenomenon, we exposed repeated generations of *C. elegans* to either 1 or 2% glucose. A 2% glucose exposure had previously been demonstrated to reduce the life span of the exposed generation (*26*–*29*) and cause a transgenerational reduction in fertility for three generations (*29*) and an intergenerational increased resistance to oxidative stress and protection from neurodegeneration (*29*). We found that *C. elegans* exposure to elevated glucose led to an increase in neutral lipids as assessed by oil red O staining (Fig. 2, A and B, and table S3) in the exposed generation. This parental exposure induced a decrease in neutral lipid content in the F1 generation (Fig. 2, C and D, and table S3), suggesting that elevated parental glucose content causes intergenerational decreased neutral lipids. Similar to what we had found with the effect of repeated hypoxia exposure, we found that repeated generational exposure to elevated glucose eliminated the second generation’s capacity to respond to elevated glucose (Fig. 2, E and F, and table S3). In addition, we found that the decrease in fertility observed in response to elevated glucose (Fig. 2G and table S4) was still present after a successive generational exposure to elevated glucose (Fig. 2H and table S4) but was eliminated in the third repeated generational exposure to elevated glucose (Fig. 2I and table S4). Together, these results suggest that *C. elegans* adapt with regard to their fat content and fertility in response to repeated exposures to elevated glucose similar to what we had observed with life span and fertility in response to repeated hypoxia exposure (Fig. 1). These findings suggest that transgenerational adaptation is a general phenomenon in response to repeated exposure to environmental stresses.

### Transgenerational adaptation to hypoxia requires genes involved in metabolism, immunity, and the stress response

**Fig. 3.**
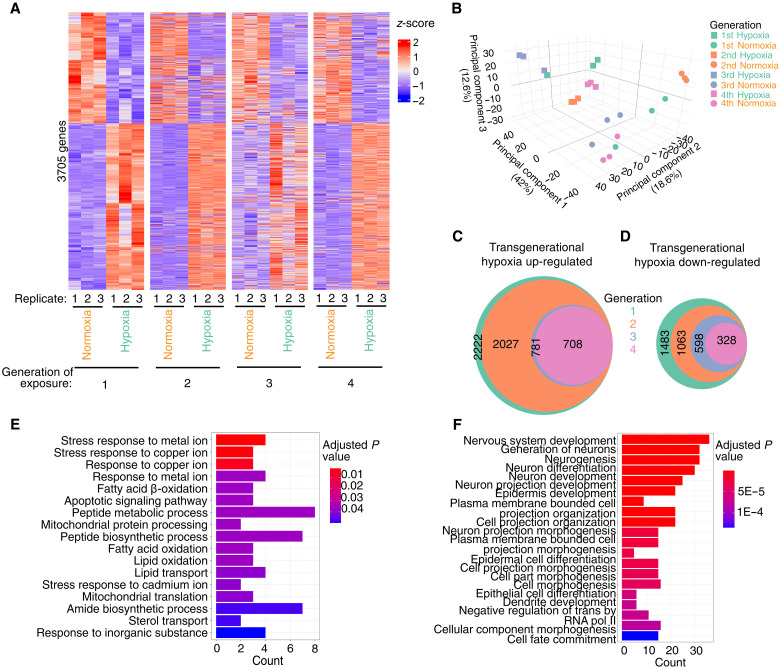
Repeated generational hypoxia exposure reveals transgenerationally adapted gene expression. (**A** and **B**) Repeated generational exposure to hypoxia induces the dysregulation of a roughly similar set of genes at each generation as visualized by (A) heatmap and (B) principal components analysis. (**C** and **D**) Venn diagrams reveal which transcripts are consistently (C) up-regulated or (D) down-regulated in response to repeated hypoxia exposure. (**E**) GO analysis of 73 consistently up-regulated genes in response to repeated hypoxia exposure in the first to third generation that are not up-regulated in the fourth generation reveals genes involved in stress response, metabolism, apoptosis signaling, as well as lipid oxidation and transport. (**F**) GO analysis of 270 consistently down-regulated genes in response to repeated hypoxia exposure in the first to third generation that are not down-regulated in the fourth generation reveals genes involved in development, organization, and cell fate determination.

To determine how *C. elegans* adapted to repeated generational exposures to hypoxia, we performed RNA sequencing (RNA-seq) of normoxia-reared controls and *C. elegans* exposed to hypoxia for one, two, three, or four generations by which point all hypoxia-induced phenotypes we tested were no longer responsive to hypoxia treatment. We found that hypoxia treatment induced similar gene expression changes as we had previously observed (fig. S1A; *r*^2^ = 0.96) (*24*), suggesting that our RNA-seq analysis and hypoxia treatment were consistent and reproducible. We found that hypoxia treatment in a single generation caused an up-regulation of 2222 genes and a down-regulation of 1483 genes, which reached our significance cutoff across multiple datasets (Fig. 3A). These genes were enriched for genes involved in a variety of metabolic processes, immune response, and lipid catabolism (fig. S2 and table S5). We found that hypoxia treatment elicited mostly similar changes in gene expression if performed for one, two, three, or four successive generations (Fig. 3, A and B, and fig. S1B). We were most interested in genes whose altered expression disappeared when the transgenerational phenotypes displayed adaptation, in the second generation for longevity and the fourth generation for fertility. These genes would include those that were dysregulated in response to hypoxia treatment after one generation but not after two, three, or four generations as these would be hypoxia-responsive genes that adapted to regulate longevity; genes that were dysregulated in response to hypoxia treatment after one, two, and three generations but not after four generations would be hypoxia-responsive genes that adapted to regulate fertility.

We found that, of the 3705 statistically significantly dysregulated genes in response to one generation of hypoxia exposure, 2027 genes were consistently up-regulated and 1063 genes were consistently down-regulated after two successive hypoxia exposures. Of these genes, 781 were consistently up-regulated and 598 were consistently down-regulated after three successive hypoxia exposures. Of these genes, 708 were consistently up-regulated and 328 were consistently down-regulated after four successive generations of hypoxia exposure (Fig. 3, C and D, and fig. S1C). We therefore focused on genes that were dysregulated when phenotypes of extended life span or reduced fertility were present but no longer dysregulated when those phenotypes disappeared (in the second and fourth generation, respectively). These comparisons revealed 195 up-regulated and 420 down-regulated genes that were dysregulated after one generation of hypoxia but not after two generations of successive hypoxia whose adapted expression are presumably involved in the intergenerational adaptation to hypoxia that regulates longevity. The adaptation genes were enriched for genes involved in sugar metabolism and immune response genes, which could potentially help to explain the extended longevity phenotype in the first but not the second generation (fig. S2, A and B). The comparisons of hypoxia-dysregulated genes in generations one, two, and three to those no longer dysregulated in generation four (the transgenerational hypoxia-adapted fertility genes) consisted of 73 up-regulated genes (Fig. 3E) and 270 down-regulated genes of interest (Fig. 3F). An analysis of these transgenerationally adapted genes revealed up-regulated genes that were enriched in apoptosis signaling, the response to different metals, as well as lipid oxidation and transport (Fig. 3E). The down-regulated transgenerationally adapted genes were enriched in development, organization, and cell fate determination genes (Fig. 3F), which might help to explain the absence of a fertility defect in the fourth generation of successive hypoxia treatments. Together, these gene expression analyses revealed that most hypoxia-responsive genes still respond to hypoxia treatment despite repeated hypoxia exposures (Fig. 3, A and B, and fig. S1B) and that only a very small number of genes displayed adaptations in terms of expression that could correlate with the adaptations of phenotypes that we observed.

To further refine our examination of how transcription changes differ in a single generation exposure to hypoxia from repeated hypoxia exposures, we performed comparisons of the gene expression dataset generated in this study to the one we had previously generated (*24*). We found that the greater majority of the 949 shared hypoxia-responsive genes present in both datasets maintained consistent dysregulation after repeated hypoxia exposure but mostly lost their dysregulation in subsequent generations in response to a single hypoxia exposure (fig. S3A). Analysis of this restricted set of hypoxia transgenerationally dysregulated genes did not reveal any major differences to the first dataset. A gene set enrichment analysis of the 134 genes that were dysregulated in the P0 but not in the F1 in response to repeated hypoxia did not reveal any obvious longevity regulating candidates (fig. S3B), although perhaps pathways involved in protein and fat binding or lipid metabolism could be involved in that hypoxia-adapted phenotype as lipid metabolism genes have been demonstrated to play a role in regulating longevity (*30*, *31*). A gene ontology (GO) analysis of the 219 significantly dysregulated genes from the first, second, and third generations of repeated hypoxia that were not dysregulated in the fourth generation again revealed genes involved in development, which could explain the defects in fertility (fig. S3C). Together, these analyses begin to help to reveal how *C. elegans* can adapt to repeated generational stresses on a transcriptional level.

### H3K27 trimethylase regulating enzymes are required for transgenerational adaptation

**Fig. 4.**
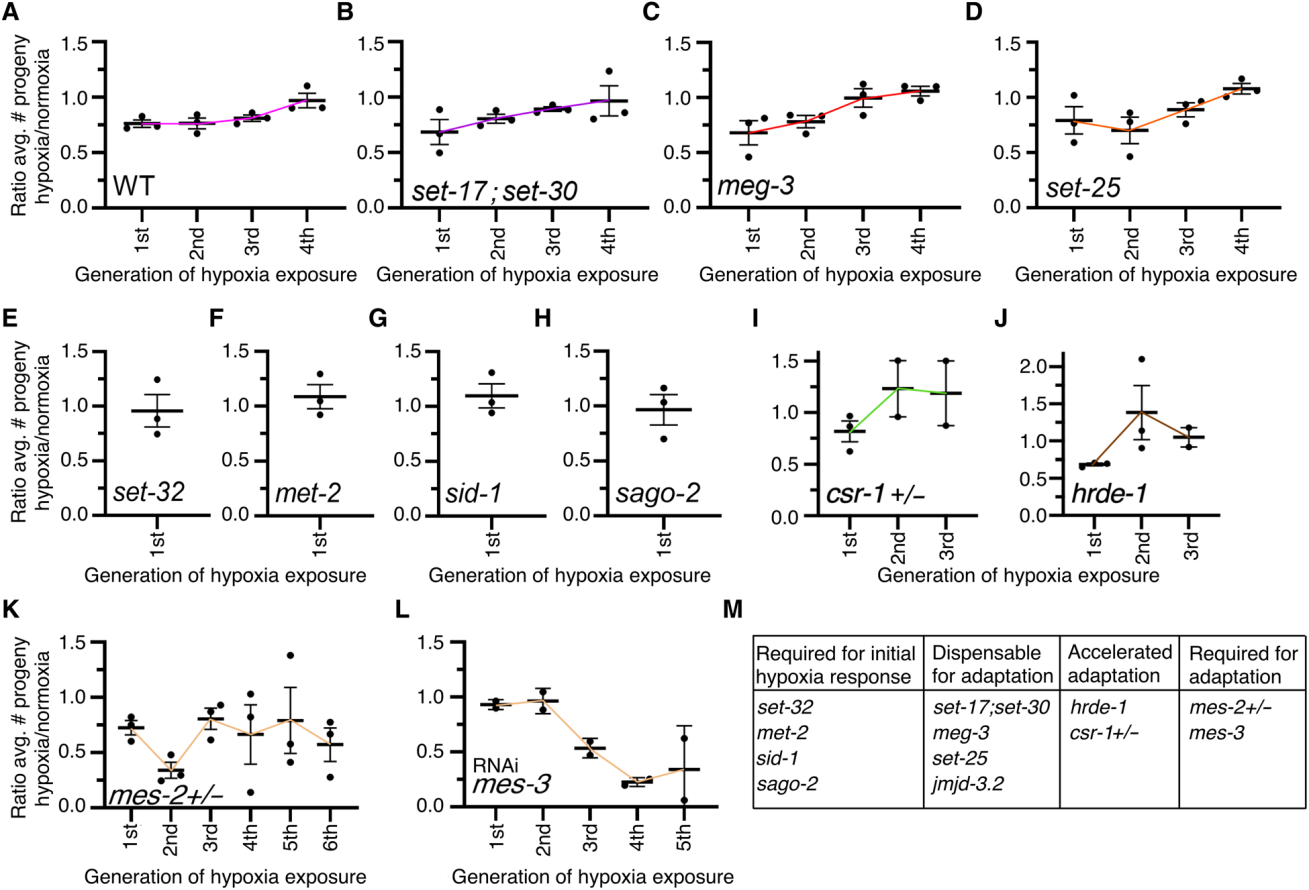
The PRC2 complex is required for transgenerational adaptation to hypoxia. (**A** to **D**) *set-17;set-30*, *meg-3*, and *set-25* mutant worms all adapted to repeated generational hypoxia exposure after four generations as WT worms do, suggesting that these genes are not involved in transgenerational adaptation to repeated hypoxia exposure. (**E** to **H**) *set-32*, *met-2*, *sid-1*, and *sago-2* mutant worms do not display reduced fertility in response to a single generation of hypoxia, suggesting that these genes are necessary for the initial hypoxia response. (**I** and **J**) *csr-1* heterozygous and *hrde-1* mutant worms adapt to repeated generational hypoxia exposure after two generations rather than four generations, suggesting that these argonaute genes are required for maintaining the generational clock. (**K** and **L**) *mes-2* heterozygous mutant worms and knockdown of *mes-3* eliminated the adaptation to repeated generational hypoxia exposure, suggesting that these genes are required for transgenerational adaptation. Each dot represents an independent experiment consisted of three replicate plates with 10 worms per plate. For *hrde-1* and *mes-2* mutant strains, 20 worms were used per plate. The ratio of the average number of eggs laid by hypoxia-treated worms divided by the average number of eggs laid by normoxia-treated worms is displayed. Error bars represent the SEM, and colored lines are used as a trendline across generations. (**M**) Summary of all genetic screening experiments.

To determine how transgenerational adaptation to hypoxia was regulated, we first examined our RNA-seq data to see whether there were any epigenetic regulatory enzymes that displayed expression patterns that would match the phenotypic observations. However, we failed to observe any epigenetic regulators that displayed dysregulation after one generation of hypoxia but not subsequent generations or after three repeated generations of hypoxia but not after the fourth (fig. S2C). Because altered expression of epigenetic regulatory enzymes did not correlate with the transgenerational adaptation phenotypes and because epigenetic regulatory enzymes are regulated in many different ways other than gene expression levels (*32*, *33*), we proceeded to perform a directed genetic screen on epigenetic regulating genes that we had previously tested for their requirements for transgenerational epigenetic inheritance in response to a single exposure to hypoxia (*24*). We identified that the H3K4 mono- and dimethyltransferases SET-17 and SET-30 (*34*), the intrinsically disordered protein MEG-3, which is involved in phase separation through RNA binding (*35*), the putative H3K27me3 demethylase JMJD-3.2 (*36*), and the putative H3K9 trimethyltransferase SET-25 (*37*) were all dispensable for transgenerational adaptation to hypoxia (Fig. 4, A to D, and fig. S3). We had previously determined that *jmjd-3.2*, *set-25*, and *meg-3* were both required for the transgenerational epigenetic inheritance of fertility defects in response to a single exposure of hypoxia (*24*). These genes are necessary for the transgenerational hypoxia response but are dispensable for transgenerational adaptation, demonstrating that these disparate phenomena are regulated by independent mechanisms.

As we had previously reported (*24*), we confirmed that the H3K23 trimethyltransferase SET-32 (*38*), the dsRNA transporter SID-1 (*39*), and the small RNA argonaute SAGO-2 (*40*) were all required for the initial response to hypoxia and expanded these findings by identifying that the putative H3K9 methyltransferase MET-2 (*41*) was also required for the initial response to hypoxia (Fig. 4, E to H), and therefore it is not possible to determine whether these genes play a role in transgenerational adaptation. We determined that deletion of the argonaute proteins, CSR-1 (*42*) and HRDE-1 (*43*), accelerated the adaptation to hypoxia (Fig. 4, I and J). Although the hypoxia-exposed *csr-1* and *hrde-1* mutant worms laid fewer eggs in the first generation, after another successive exposure to hypoxia, *csr-1* and *hrde-1* mutant worms laid comparable or even slightly higher numbers of eggs to normoxia worms (Fig. 4, I and J). We had previously found that *hrde-1* was required for transmitting the transgenerational epigenetic inheritance of fertility defects in response to a single exposure of hypoxia (*24*), suggesting that small RNAs are involved in both the transgenerational hypoxia response and the transgenerational adaptation process.

We identified that worms that were heterozygous for the putative H3K27 trimethyltransferase *mes-2* (*44*) never adapted to repeated successive hypoxia exposures (Fig. 4K). MES-2 is the catalytic enzyme of a multisubunit protein complex, termed the polycomb repressive complex 2 (PRC2), that is important for maintaining repressive H3K27me3 chromatin modifications (*45*–*48*). We had previously found that *mes-2* was also required for inheritance in response to a single generation exposure to hypoxia (*24*). MES-3 is an ortholog of the suppressor of Zeste 12 (SUZ12) (*44*, *49*), a core subunit of the PRC2 complex that provides a structural scaffold for the complex and is necessary for the methyltransferase activity (*50*, *51*). *mes-3* knockdown also prevented adaptation to repeated successive hypoxia exposures (Fig. 4L). *mes-3* knockdown in conjunction with hypoxia treatment made the fertility relative to normoxia virtually indistinguishable until the third generation. This finding could be due to incomplete penetrance of RNA interference (RNAi), a resetting of exogenous heritable dsRNA (*52*), or a synergism between the maternal effect sterile phenotype of *mes-3* depletion and hypoxia treatment having altered penetrance over successive generations. Together, however, these results suggest that this complex and H3K27 methylation are required for the transgenerational adaptation to hypoxia.

## DISCUSSION

Through these experiments, we showed that *C. elegans* adapt on a transgenerational scale to repeated hypoxia and elevated glucose exposures. We found that, after two generations of successive hypoxia exposure, *C. elegans* no longer display a hypoxia-induced life-span extension (Fig. 1D). We also found that, after four generations of successive hypoxia exposure, *C. elegans* no longer display a hypoxia-induced fertility defect (Fig. 1B). This phenomenon was not specific to hypoxia exposure as repeated generation exposure to elevated glucose also caused an adaptation in *C. elegans* (Fig. 2). We observed that, for the most part, repeated hypoxia exposure continued to cause the dysregulation of many transcripts (Fig. 3A and fig. S1B). However, we identified a subset of transgenerationally adapted genes whose expression was no longer altered in response to two successive exposures to hypoxia and were involved in sugar metabolism and immune response genes, raising the possibility that these genes could be important for life-span regulation in response to hypoxia. Similarly, we identified a subset of transgenerationally adapted genes whose expression was no longer altered in response to four successive exposures to hypoxia and were involved in apoptosis signaling, the response to different metals, as well as lipid oxidation and transport, raising the possibility that these genes are important for fertility regulation. Through a directed genetic screen, we found that the argonautes CSR-1 and HRDE-1 as well as the H3K27 trimethyltransferase complex are necessary for transgenerational adaptation to hypoxia. Together, these results demonstrate that organisms can adapt on a transgenerational scale to repeated stresses and that this transgenerational adaptation shares some of the same epigenetic regulating enzymes that are required for the initial transgenerational response to that same stress.

Our previous work had identified that both HRDE-1 and MES-2 were necessary for the transgenerational response to a single generational exposure to hypoxia (*24*). When these genes were deleted, *C. elegans* displayed an initial response to hypoxia but were unable to transmit the phenotypes to naïve normoxia-reared descendants (*24*). Here, we found that *hrde-1* deletion and ablation of the PRC2 complex had opposite phenotypic consequences. Deletion of *hrde-1* accelerated the transgenerational adaptation, such that two successive generations of hypoxia exposure was sufficient to induce transgenerational adaptation instead of four repeated generations (Fig. 4J), whereas ablation of the PRC2 complex made it so that worms were resistant to transgenerational adaptation (Fig. 4, K and L). Although these findings are mostly phenomenological in their essence, they do point to some exciting potential mechanisms that could be further followed up on with regard to small RNAs and H3K27me3 inheritance. It is possible that one set of small RNAs could be responsible for an initial transgenerational response whereas a second set of small RNAs could be responsible for the transgenerational adaptation response. Similarly, H3K27me3 could mark the histones of one set of genes important for an initial transgenerational response and a second set of genes important for the transgenerational adaptation response, and both sets of genes and small RNAs could independently be important for conferring extended longevity and reduced fertility.

Many, but not all, of the genes we identified as being necessary for transgenerational epigenetic inheritance in response to a single exposure of hypoxia (*24*) were also involved in the transgenerational adaptation to repeated hypoxia exposure. Because the transgenerational epigenetic inheritance and the transgenerational adaptation to hypoxia are almost two opposing phenomena, it is perhaps expected that small RNA regulation is necessary for the former and deletion accelerates the latter. However, ablation of PRC2 inhibits both transgenerational epigenetic inheritance and transgenerational adaptation from occurring. This finding harkens back to the opposing role of EZH2 as an oncogene or a tumor suppressor (*53*–*56*) and a pro-aging and pro-longevity signal depending on its context (*57*–*59*), highlighting the context-dependent nature of epigenetic regulators. This reinforces that these enzymes may have alternative functions in different cell types or circumstances. It will be interesting, in future studies, to investigate how H3K27me3 regulation can accomplish these opposing actions in transgenerational epigenetic inheritance.

Unexpectedly, the transgenerational adaptation of different traits, such as life span and fertility, took place on a different generational time scale, with longevity adapting after two successive generations but fertility requiring four to fully adapt. This difference suggests that either the two different traits have different thresholds for altered phenotypes and there is simply a slow decrease in the heritable molecules, which happens to pass the threshold for longevity before the threshold for altering fertility occurs, or the environmental changes are inducing many independent signaling pathways in parallel and some are no longer induced in response to repeated hypoxia more rapidly than other signaling pathways. These two options are not mutually exclusive, and it will be interesting, in future experiments, to identify the universe of all heritable materials and identify which molecules are specifically responsible for which heritable phenotype.

We had also previously identified that a single generation of hypoxia exposure caused an intergenerational reduction of neutral lipids (*24*). In this current study, we found that our GO analysis for transgenerationally adapted up-regulated genes revealed an enrichment for genes involved in lipid oxidation and lipid transport. Given that some lipids function as signaling molecules between cells (*60*) and between organisms (*61*) and even potentially across generations (*62*, *63*), it will be interesting to determine whether altered lipid levels are simply a consequence of hypoxia treatment or could be involved in transmitting nongenetic information across generations.

Transgenerational epigenetic inheritance has always functioned at the interface between genetics and environment, allowing organisms to adapt on a relatively short time scale, from an evolutionary perspective, of a few generations. This phenomenon allows organisms to inform the subsequent generations that conditions might not be optimal and they should therefore adapt to prepare for such an eventuality. Transgenerational adaptation, on the other hand, would exist if such a detrimental environmental condition were perpetually present; the organism would not want to persist in a heightened adapted condition but would instead accept that these were the new conditions for the foreseeable future in their domain. It will be exciting to determine whether transgenerational adaptation is a prevalent phenomenon, occurring in response to many environmental stressors that induce transgenerational epigenetic inheritance, or whether this is a unique responder to hypoxia and glucose. If this phenomenon is present in response to multiple stresses, it will be interesting to investigate whether each instance of transgenerational adaptation is dependent on small RNAs and H3K27me3 regulating enzymes or, if like transgenerational epigenetic inheritance itself, these transgenerational adaptations function through multiple independent parallel epigenetic regulatory mechanisms.

## MATERIALS AND METHODS

### Experimental design

The objectives of this study are to understand how *C. elegans* respond to repeated generational exposure to stressors such as hypoxia and glucose.

#### Hypoxia exposure

The worms were kept at 20°C and were well fed before exposure. L4 worms were transferred to a new Nematode Growth Media (NGM) plate with sufficient food and were exposed to a 0.1% oxygen level for 16 hours using an Eppendorf Galaxy 48R incubator. After 16 hours, they were moved back to normal atmospheric oxygen level (~20% O_2_). Normoxia/control worms remained in an incubator at normal oxygen levels for the entire experiment. The progeny of the first generation were selected at the L4 stage, and this process was repeated across multiple generations (Fig. 1A).

#### Glucose exposure

Glucose (1 or 2%) was added in NGM plates seeded with OP50. Eggs were put onto plates after bleaching and allowed to grow til day 1 of adulthood at 20°C. For subsequent generations, 20 to 30 worms were transferred onto fresh OP50 with or without glucose supplementation and allowed to lay eggs for 2 hours. The mother worms were then euthanized. In each generation, oil red O was performed at the post–young adult stage.

### *C. elegans* strains

The N2 Bristol strain was used as the wild-type (WT) background. The following mutations were used in this study: *hrde-1(tm1200)*, *csr-1(tm892)* IV/nT1 [unc-?(n754) let-?] (IV;V), *mes-3(bn21)*, *jmjd-3.1(gk384)*, *jmjd-3.1(gk387)*, *jmjd-3.2(tm3121)*, *meg-3(tm4259)*, *mes-2(bn11) unc-4(e120)/mnC1 [dpy-10(e128) unc-52(e444)]*, *met-2(ok2307)*, *sago-2(tm894)*, *set-17(n5017)/set-30(gk315)*, *set-25(n5021)*, *set-32(ok1457)*, and *sid-1(qt9)*. Worms were grown on OP50 bacteria in all experiments.

### Antibodies

Antibodies used for CUT&RUN were rabbit anti-H3 (Abcam, Ab1791) and rabbit anti-H3K27me3 (MilliporeSigma, 07-449). These antibodies have demonstrated specificity in eukaryotes. The CUT&RUN experiment was unsuccessful due to technical difficulties.

### Fertility assay

NGM plates with OP50 were prepared as described above. Ten worms at the L4 stage were transferred to OP50-seeded NGM plates in triplicate (30 worms per condition) and kept at 20°C. The worms were transferred to new plates every day for 2 days. The number of hatched worms was counted.

### Mes-3 RNAi fertility assay

Preparation of *mes-3* RNAi petri plates: 20 g of agar, 3 g of NaCl, and 5 g of Bacto Peptone was added to a 2-liter flask with 1 liter of deionized H_2_O and autoclaved for 55 min on liquid cycle. The flask was cooled to ~55°C before adding in 25 ml of 1 M KPO_4_ buffer, 1 ml of 1 M MgSO_4_ buffer, 1 ml of 1 M CaCl_2_ buffer, 1 ml of cholesterol (5 mg/ml) dissolved in ethanol, 0.4 mM IPTG (isopropyl-β-d-thiogalactopyranoside), and 0.5 ml of ampicillin (3 mg/ml). Ten milliliters of this mixture was added to each petri dish (60 mm by 15 mm) and left at room temperature for 24 hours to solidify. Two hundred microliters of F54C1.3 bacteria was added for the *mes-3* plates and HT115(DE3) bacteria for the empty vector control. L4 worms were transferred from OP50 NGM plates to their respective plates. The hypoxia exposure step and fertility assay were conducted as described above.

### Longevity assay

Worm life-span assays were conducted at 20°C, and worms were grown on OP50 NGM plates without 5-fluoro-2′-deoxyuridine (FUdR). For each assay, roughly 90 worms per condition were observed on three plates (30 worms per plate) at the beginning of the experiment. Worms that crawled off the plate, had a ruptured vulva, or experienced matricide were censored. Kaplan-Meier curves were plotted and visualized in GraphPad Prism. *P* values were calculated using log-rank tests.

### RNA sequencing

#### C. elegans *age synchronization*

Plates with worms were synchronized by washing the adult worms off the plates using M9 buffer with 1% Triton X-100 once a sufficient number of eggs were laid. Through gentle pipetting, the worms are washed away whereas the eggs remain intact on the bacterial lawn. These eggs were reared at 20°C and hatched. Once most worms appeared to be at the L4 stage, they were exposed to either normoxia or hypoxia as per the experimental setup.

#### C. elegans *sample collection*

Because this experiment focuses on multiple generations of *C. elegans*, it was important to collect and freeze worm samples so that they could be used for the following experiments simultaneously. Three biological replicates for each condition between the first and fourth generations were produced, and a total of 24 samples were collected. After the 16-hour exposure to normoxia or hypoxia, worms were washed off the plate using M9 buffer with 1% Triton X-100 and collected in a 1.5-ml microcentrifuge tube. The worms were allowed to settle to the bottom, and the excess buffer was removed. The worm pellet was then flash frozen in liquid nitrogen for 5 s and stored at −80°C until use.

#### Total RNA extraction for RNA-seq

Total RNA was extracted using the Direct-zol RNA Miniprep Kit from Zymo Research. Briefly, frozen worm samples were lysed by adding in 300 μl of TRI Reagent and homogenizing with a Kontes pellet pestle motor for 1 min. An equal amount of ethanol was added and mixed thoroughly before being transferred to a Zymo-Spin IICR Column in a collection tube and centrifuged. The column matrix was treated with DNase I and DNA Digestion Buffer and incubated at room temperature for 15 min to get rid of any unwanted traces of DNA. Afterward, the column was washed twice with Direct-zol RNA PreWash and once with RNA Wash Buffer. The column was then transferred in a clean tube, and the RNA was eluted by adding 25 μl of DNase/RNase-Free Water to the column matrix and centrifuged for 30 s. The RNA concentration and purity were measured using a NanoDrop before storing at −80°C until use.

#### RNA-seq library preparation

mRNA was enriched from total RNA samples using NEXTFLEX Poly(A) Beads 2.0. Libraries for RNA-seq with these samples were prepared using the NEXTFLEX Rapid Directional RNA-Seq Kit 2.0. Twenty-four samples were prepared, each with 1 μg of the mRNA starting material. In short, mRNA is fragmented before first and second strand synthesis. Strands are then adenylated, and index adapters are ligated, followed by 11 cycles of polymerase chain reaction (PCR) amplification. The PCR products were cleaned up using NEXTFLEX Cleanup Beads XP and transferred to nonstick RNase-free microcentrifuge tubes and stored at −20°C until sample submission. RNA quality was validated using Agilent 2200 TapeStation D1000 before being sequenced using a NovaSeq 6000 SP platform.

Fastq files were checked for quality using FastQC before being filtered out with a *Q* score of 30 or above and trimmed for adapters using CutAdapt 4.0. The trimmed reads were then aligned to the *C. elegans* genome (WS235) using bowtie2, and samtools was used to convert sam outputs to sorted bam files. FeatureCounts was used to quantify the number of counts for each gene using the sorted bam files. Genes under 350 counts were filtered out, and DESeq2 was used to normalize raw counts. Differentially expressed genes with a *P* value of > 0.05 was filtered out before downstream analyses. RNA-seq figures were plotted using ggplot2, ComplexHeatMap, clusterProfiler, AnnotationDbi, org.Ce.eg.db, and EnhancedVolcano packages in RStudio.

### Oil red O staining

Eggs were grown in NGM OP50 with or without 1 or 2% glucose addition. Day 1 adults were stained with oil red O as previously described (*64*). Worms were mounted on a glass slide with an agarose pad [2% (w/v) agarose in ddH_2_O] and visualized under a bright-field microscope (Leica Dmi8). The entire worm oil red O staining was quantified using ImageJ Fiji 5.

### Statistical analysis

The number of replicates, *P* values, and statistical test used for each experiment is indicated in the corresponding figure legend and detailed in this section. *P* values for independent replicates of fertility assays (Fig. 1B) were determined by two-tailed *t* test. *P* values for Kaplan-Meier curves (Fig. 1, C and D) were calculated using log-rank rests (Mantel-Cox), and *P* values for glucose exposure assays (Fig. 2, A, C, E, G, H, and I) were determined using one-way analysis of variance (ANOVA). *P* values for independent replicates were combined using combined Fisher’s test. The *r*^2^ value quantifying the relationship between the logFC (log fold change) values of significant genes in the P0 generation for this study and previously observed differentially expressed genes from hypoxia treatment (*24*) was derived from a standard linear regression model (fig. S1A).
