# Supplementary material for: Transgenerational adaptation to hypoxia
Source: Sci Adv. 2025 Oct 24;11(43):eadv9451. doi: 10.1126/sciadv.adv9451 (PMC12551719; doi:10.1126/sciadv.adv9451)
Supplement: Supplementary file 1 — Figs. S1 to S4 Tables S1 to S4 Legend for table S5 [file sciadv.adv9451_sm.pdf]

Science Advances
AAAS

Supplementary Materials for

Transgenerational adaptation to hypoxia

Kathleen Kim *et al.*

Corresponding author: Simon Yuan Wang, simonwang602@gmail.com; Eric Lieberman Greer, ericg@wustl.edu

Sci. Adv. **11**, eadv9451 (2025)
DOI: 10.1126/sciadv.adv9451

The PDF file includes:

Figs. S1 to S4
Tables S1 to S4
Legend for table S5

Other Supplementary Material for this manuscript includes the following:

Table S5

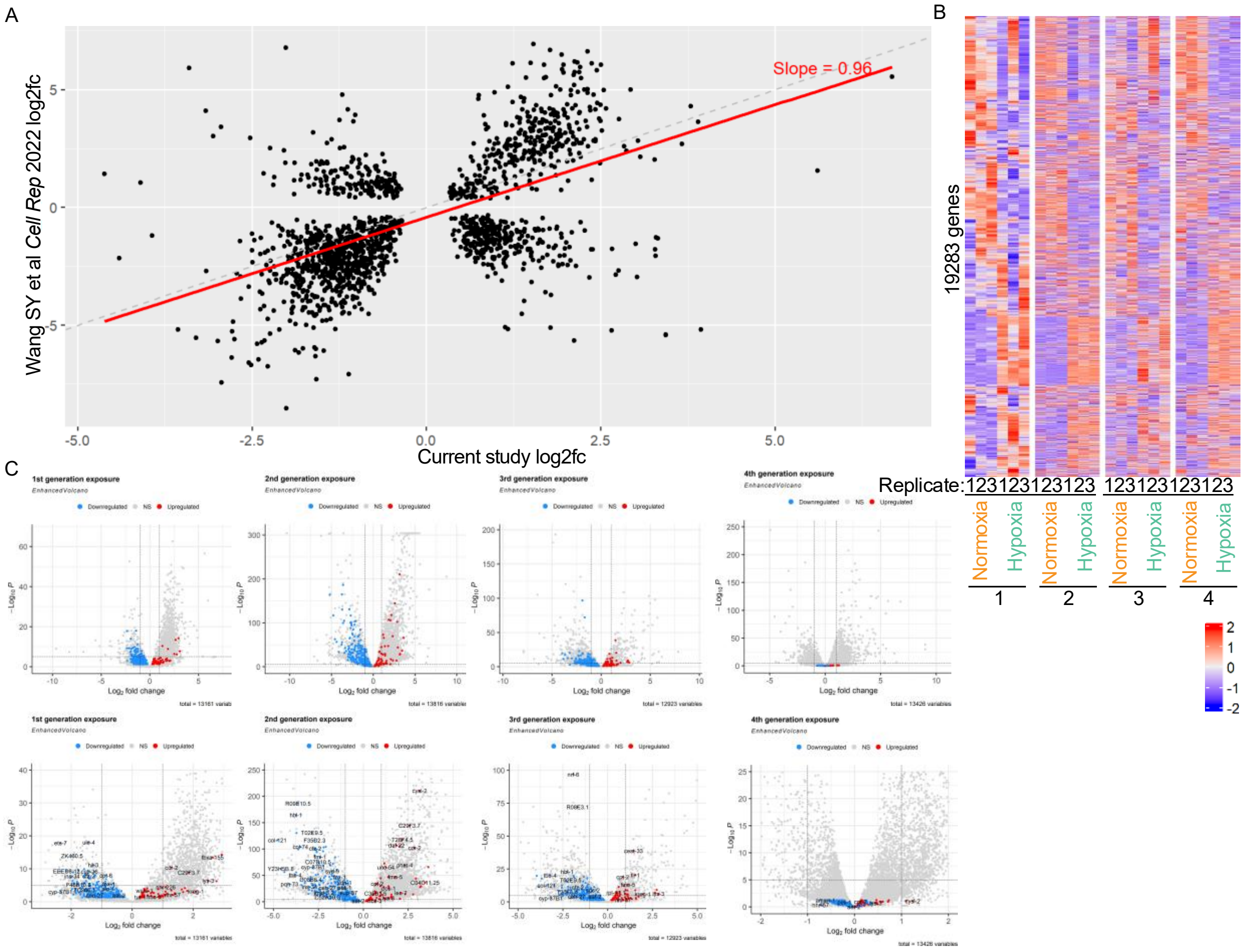

Fig. S1. Repeated generational hypoxia exposure reveals transgenerationally adapted gene expression (**A**) Comparison of P0 generation hypoxia treated worms in the current study to hypoxia treated worms for a single generation from our previous manuscript (24) revealed a very similar set of genes were dysregulated upon hypoxia treatment ($r^2 = 0.96$), suggesting that our RNA sequencing analysis and hypoxia treatment were consistent and reproducible. (**B**) A heatmap of 19283 genes after being filtered out for low read counts was generated across all 4 exposures with all three biological replicates included using ComplexHeatMap package. (**C**) Volcano plots reveal hypoxia relative to normoxia treated controls in each generation with the transgenerationally significant genes shown in red (upregulated) or blue (downregulated) to reveal that the directionality of the changes in gene expression is similar from generation to generation.

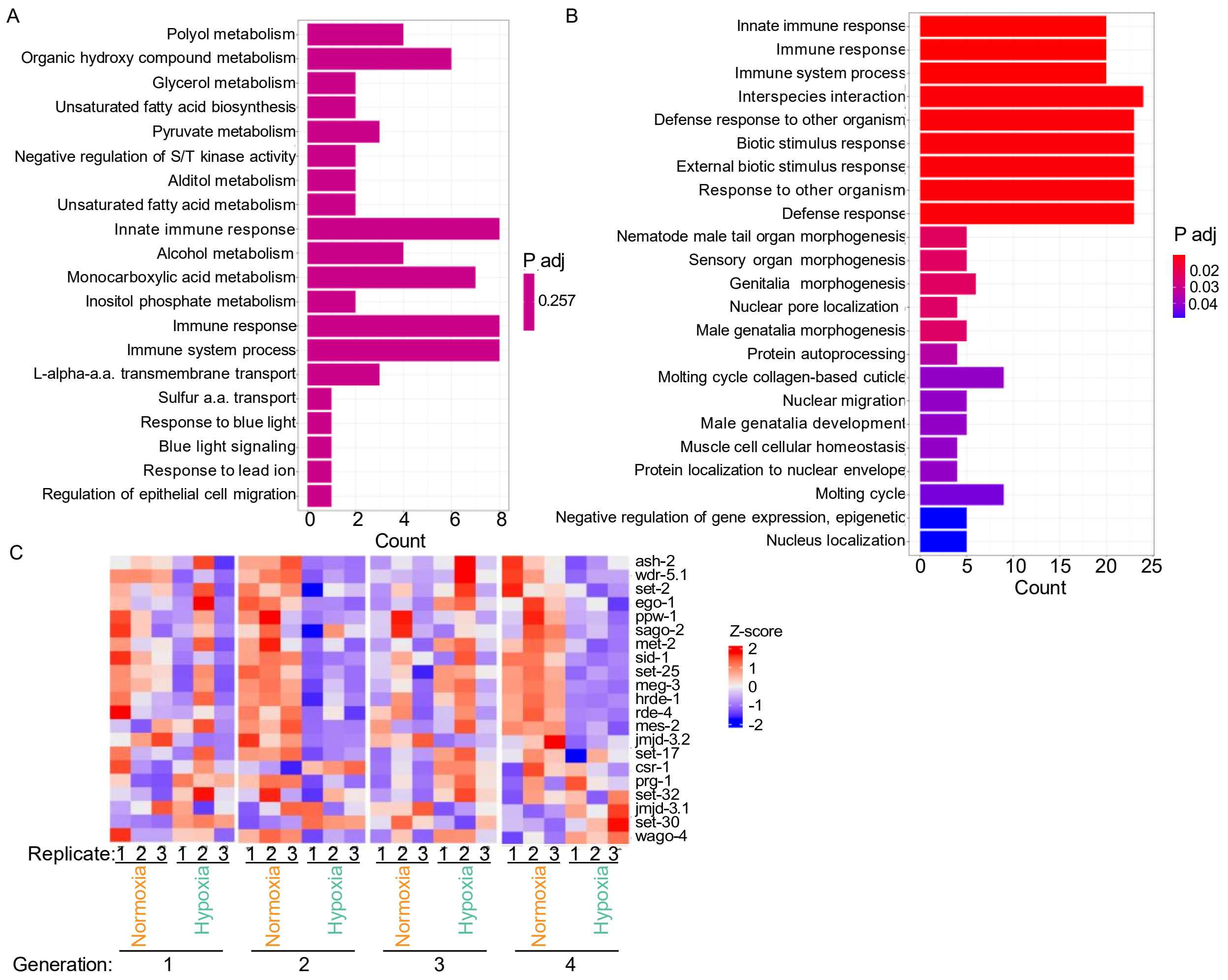

Fig. S2. Transgenerational adaptation genes after 2 successive generations of hypoxia exposure are enriched for sugar metabolism and immune response genes and epigenetic regulator enzymes do not reveal adapted gene expression. (**A**) GO analysis of 195 upregulated genes in response to single hypoxia exposure in 1st generation that are not upregulated in the 2nd successive hypoxia exposed generation reveals genes involved the immune response and metabolism. (**B**) GO analysis of 420 downregulated genes in response to a single hypoxia exposure in 1st generation that are not downregulated in the 2nd successive hypoxia exposed generation reveals genes involved in the immune response and development. (**C**) A heat map of gene expression of epigenetic regulators across four successive generations of hypoxia exposure did not reveal adaptation of known epigenetic regulator gene expression.

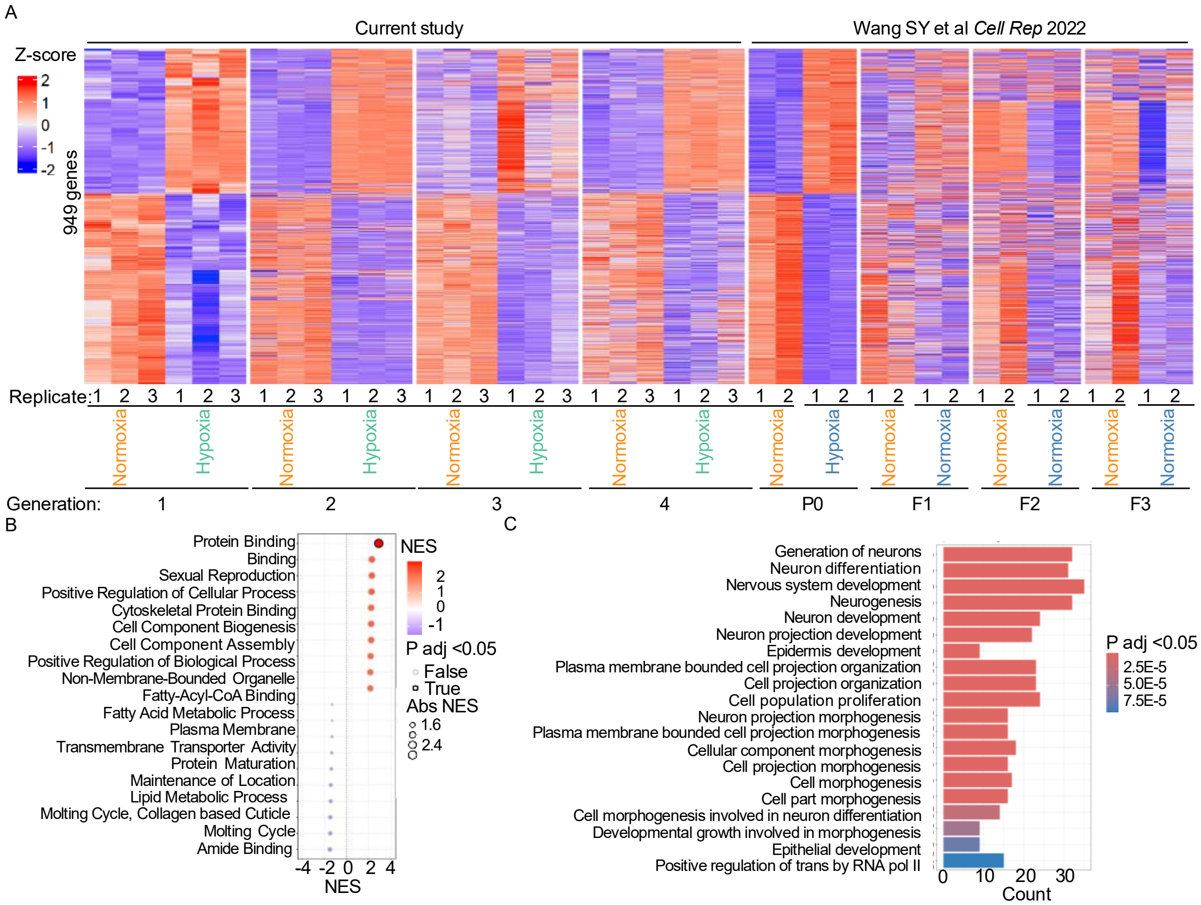

Fig. S3. Transgenerational adaptation compared with transgenerational epigenetic inheritance in response to hypoxia exposure (**A**) A heat map of the 949 shared hypoxia dysregulated genes across this study and our previous work (24) reveals that while the greater majority of hypoxia dysregulated genes lose dysregulation after a single generation exposure to hypoxia most hypoxia responsive genes maintain dysregulation in response to repeated generational hypoxia exposure. (**B**) GSEA analysis 134 dysregulated genes in response to a single hypoxia exposure in 1st generation that are not dysregulated in the 2nd successive hypoxia exposed generation reveals genes involved in protein and fat binding and lipid metabolism. (**C**) GO analysis of 219 dysregulated genes from the 1st, 2nd, and 3rd generations of repeated hypoxia treatment that are no longer dysregulated in the 4th generation of repeated hypoxia are enriched for genes involved in development.

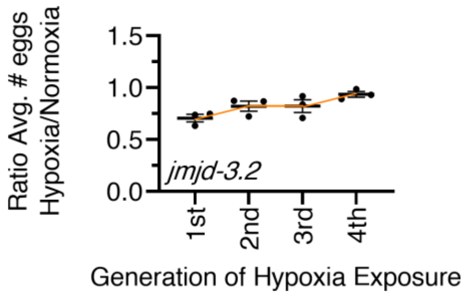

Fig. S4. The putative H3K27me3 demethylase JMJD-3.2 is dispensable for transgenerational adaptation to hypoxia. *Jmjd-3.2* mutant worms adapted to repeated generational hypoxia exposure after 4 generations as WT worms do suggesting that *jmjd-3.2* is not involved in transgenerational adaptation to repeated hypoxia exposure. Each dot represents an independent experiment consisted of three replicate plates with 10 worms per plate. The ratio of average number of eggs laid by hypoxia-treated worms divided by the average number of eggs laid by normoxia-treated worms is displayed. Error bars represent the SEM and colored lines are used as a trendline across generations.

Strain + replicate	Generation	Condition	Mean +/- SD	P values
WT – replicate 1	1st Exposure	Normoxia	271.20 +/- 12.48	
WT – replicate 1	1st Exposure	Hypoxia	234.10 +/- 10.02	0.0159
WT – replicate 1	2nd Exposure	Normoxia	344.60 +/- 32.15	
WT – replicate 1	2nd Exposure	Hypoxia	287.67 +/- 49.49	0.1701
WT – replicate 1	3rd Exposure	Normoxia	297.90 +/- 55.91	
WT – replicate 1	3rd Exposure	Hypoxia	261.02 +/- 21.26	0.3457
WT – replicate 1	4th Exposure	Normoxia	267.17 +/- 48.61	
WT – replicate 1	4th Exposure	Hypoxia	257.01 +/- 54.13	0.8208
WT – replicate 1	5th Exposure	Normoxia	248.53 +/- 18.14	
WT – replicate 1	5th Exposure	Hypoxia	297.07 +/- 37.61	0.1144
WT – replicate 2	1st Exposure	Normoxia	274.67 +/- 18.90	
WT – replicate 2	1st Exposure	Hypoxia	205.33 +/- 14.09	0.0070
WT – replicate 2	2nd Exposure	Normoxia	310.80 +/- 16.24	
WT – replicate 2	2nd Exposure	Hypoxia	241.16 +/- 34.74	0.0347
WT – replicate 2	3rd Exposure	Normoxia	285.60 +/- 9.03	
WT – replicate 2	3rd Exposure	Hypoxia	263.62 +/- 33.04	0.3287
WT – replicate 2	4th Exposure	Normoxia	305.06 +/- 59.95	
WT – replicate 2	4th Exposure	Hypoxia	256.14 +/- 18.00	0.2472
WT – replicate 2	5th Exposure	Normoxia	262.13 +/- 25.75	
WT – replicate 2	5th Exposure	Hypoxia	316.27 +/- 47.60	0.1582
WT – replicate 3	1st Exposure	Normoxia	253.19 +/- 31.86	
WT – replicate 3	1st Exposure	Hypoxia	188.95 +/- 2.49	0.0253
WT – replicate 3	2nd Exposure	Normoxia	316.82 +/- 14.12	
WT – replicate 3	2nd Exposure	Hypoxia	257.63 +/-71.03	0.2298
WT – replicate 3	3rd Exposure	Normoxia	277.00 +/- 25.26	
WT – replicate 3	3rd Exposure	Hypoxia	228.50 +/- 44.46	0.1758
WT – replicate 3	4th Exposure	Normoxia	237.84 +/- 37.76	
WT – replicate 3	4th Exposure	Hypoxia	231.79 +/- 28.48	0.8353
WT – replicate 3	5th Exposure	Normoxia	228.00 +/- 90.00	
WT – replicate 3	5th Exposure	Hypoxia	244.00 +/- 53.42	0.8043
WT – replicate 4	1st Exposure	Normoxia	361.59 +/- 28.62	
WT – replicate 4	1st Exposure	Hypoxia	240.92 +/- 31.78	0.0081
WT – replicate 4	2nd Exposure	Normoxia	309.68 +/- 48.20	
WT – replicate 4	2nd Exposure	Hypoxia	237.32 +/- 31.44	0.0950
WT – replicate 4	3rd Exposure	Normoxia	287.80 +/- 13.66	
WT – replicate 4	3rd Exposure	Hypoxia	259.11 +/- 25.56	0.1616
WT – replicate 4	4th Exposure	Normoxia	265.73 +/- 44.66	
WT – replicate 4	4th Exposure	Hypoxia	286.28 +/- 33.33	0.5578
WT – replicate 4	5th Exposure	Normoxia	310.13 +/- 19.47	
WT – replicate 4	5th Exposure	Hypoxia	241.60 +/- 28.88	0.0271
WT – replicate 5	1st Exposure	Normoxia	194.47 +/- 17.15	
WT – replicate 5	1st Exposure	Hypoxia	132.47 +/- 30.74	0.0380
WT – replicate 5	2nd Exposure	Normoxia	232.27 +/- 13.67	
WT – replicate 5	2nd Exposure	Hypoxia	194.38 +/- 15.73	0.0345
WT – replicate 5	3rd Exposure	Normoxia	183.52 +/- 14.41	
WT – replicate 5	3rd Exposure	Hypoxia	145.14 +/- 12.28	0.0247
WT – replicate 5	4th Exposure	Normoxia	208.93 +/- 32.16	
WT – replicate 5	4th Exposure	Hypoxia	231.69 +/- 9.65	0.3055
WT – replicate 5	5th Exposure	Normoxia	179.00 +/- 22.86	
WT – replicate 5	5th Exposure	Hypoxia	193.27 +/- 5.80	0.3540
WT – compiled	1st Exposure	Normoxia		
WT – compiled	1st Exposure	Hypoxia		7.90e-07
WT – compiled	2nd Exposure	Normoxia		
WT – compiled	2nd Exposure	Hypoxia		0.00149
WT – compiled	3rd Exposure	Normoxia		
WT – compiled	3rd Exposure	Hypoxia		0.01728
WT – compiled	4th Exposure	Normoxia		
WT – compiled	4th Exposure	Hypoxia		0.64766
WT – compiled	5th Exposure	Normoxia		
WT – compiled	5th Exposure	Hypoxia		0.0514

Table S1 Repeated generational hypoxia exposure reduces fertility for 3 generations but does not reduce fertility in generations 4 and 5. Last row consists of compiled p-values from the three experiments using Combined Fisher's Test.

Strain	Generation	Condition	Mean +/- SD	Median	p values	# worms	Figure
WT	1st Exposure	Normoxia	15.3 +/- 0.580	17		74/87	
WT	1st Exposure	Hypoxia	17.1 +/- 0.508	19	0.087	60/85	
WT	2nd Exposure	Normoxia	15.5 +/- 0.397	14		75/83	
WT	2nd Exposure	Hypoxia	15.5 +/- 0.594	14	0.8	66/90	
WT	3rd Exposure	Normoxia	16.3 +/- 0.832	17		62/96	
WT	3rd Exposure	Hypoxia	18.5 +/- 0.604	19	0.09	64/87	
WT	1st Exposure	Normoxia	17.6 +/- 0.428	18		54/90	B
WT	1st Exposure	Hypoxia	19.9 +/- 0.734	20	0.0065	49/98	B
WT	2nd Exposure	Normoxia	17.5 +/- 0.445	19		61/107	B
WT	2nd Exposure	Hypoxia	18.0 +/- 0.529	19	0.13	65/114	B
WT	3rd Exposure	Normoxia	18.0 +/- 0.447	18		82/121	B
WT	3rd Exposure	Hypoxia	18.9 +/- 0.555	18	0.17	76/114	B
WT	1st Exposure	Normoxia	17.6 +/- 0.555	18		69/77	
WT	1st Exposure	Hypoxia	19.1 +/- 0.650	18	0.14	49/75	
WT	2nd Exposure	Normoxia	18.2 +/- 0.718	18		67/82	
WT	2nd Exposure	Hypoxia	20.3 +/- 0.885	20	0.065	44/70	
WT	3rd Exposure	Normoxia	19.4 +/- 0.750	18		73/87	
WT	3rd Exposure	Hypoxia	18.4 +/- 0.768	16	0.47	65/86	
WT compiled	1st Exposure	Normoxia					
WT compiled	1st Exposure	Hypoxia			0.0022		
WT compiled	2nd Exposure	Normoxia					
WT compiled	2nd Exposure	Hypoxia			0.1495		
WT compiled	3rd Exposure	Normoxia					
WT compiled	3rd Exposure	Hypoxia			0.0855		

Table S2 Repeated generational hypoxia exposure does not extend the lifespan of subsequent generations. Mean and median survival time was calculated with survival and survminer packages. Last row consists of compiled p-values from the three experiments using Combined Fisher's Test.

Strain + replicate	Generation	Condition	Mean +/- SEM	P values
WT – replicate 1	P0 – P0 exposure	Control	115.9 +/- 3.225	
WT – replicate 1	P0 – P0 exposure	1% glucose	128.4 +/- 2.094	0.0014
WT – replicate 1	P0 – P0 exposure	2% glucose	122.1 +/- 1.613	0.1345
WT – replicate 1	F1– P0 exposure	Control	91.02 +/- 2.577	
WT – replicate 1	F1– P0 exposure	1% glucose	79.68 +/- 2.706	0.002
WT – replicate 1	F1– P0 exposure	2% glucose	66.67 +/- 1.449	<0.0001
WT – replicate 1	F1– P0+F1 exposure	Control	91.02 +/- 2.577	
WT – replicate 1	F1– P0+F1 exposure	1% glucose	85.47 +/- 1.547	0.1202
WT – replicate 1	F1– P0+F1 exposure	2% glucose	80.24 +/- 1.755	0.0003
WT – replicate 2	P0 – P0 exposure	Control	53.02 +/- 1.805	
WT – replicate 2	P0 – P0 exposure	1% glucose	64.9 +/- 1.788	<0.0001
WT – replicate 2	P0 – P0 exposure	2% glucose	64.62 +/- 1.58	<0.0001
WT – replicate 2	F1– P0 exposure	Control	60.19 +/- 1.396	
WT – replicate 2	F1– P0 exposure	1% glucose	57.31 +/- 1.292	0.2589
WT – replicate 2	F1– P0 exposure	2% glucose	61.56 +/- 1.524	0.7152
WT – replicate 2	F1– P0+F1 exposure	Control	70.16 +/- 1.209	
WT – replicate 2	F1– P0+F1 exposure	1% glucose	69.11 +/- 1.596	0.8073
WT – replicate 2	F1– P0+F1 exposure	2% glucose	71.43 +/- 1.239	0.7406
WT – replicate 3	P0 – P0 exposure	Control	52.77 +/- 1.549	
WT – replicate 3	P0 – P0 exposure	1% glucose	79.57 +/- 1.438	<0.0001
WT – replicate 3	P0 – P0 exposure	2% glucose	70.65 +/- 1.248	<0.0001
WT – replicate 3	F1– P0 exposure	Control	88.22 +/- 1.656	
WT – replicate 3	F1– P0 exposure	1% glucose	78.25 +/- 1.534	0.0003
WT – replicate 3	F1– P0 exposure	2% glucose	79.28 +/- 3.4	0.0105
WT – replicate 3	F1– P0+F1 exposure	Control	69.54 +/- 1.403	
WT – replicate 3	F1– P0+F1 exposure	1% glucose	68.32 +/- 2.158	0.8386
WT – replicate 3	F1– P0+F1 exposure	2% glucose	73.26 +/- 1.549	0.2441
WT – compiled	P0 – P0 exposure	Control		
WT – compiled	P0 – P0 exposure	1% glucose		4.736E-9
WT – compiled	P0 – P0 exposure	2% glucose		3.094E-7
WT – compiled	F1– P0 exposure	Control		
WT – compiled	F1– P0 exposure	1% glucose		2.168E-5
WT – compiled	F1– P0 exposure	2% glucose		3.854E-5
WT – compiled	F1– P0+F1 exposure	Control		
WT – compiled	F1– P0+F1 exposure	1% glucose		0.5416
WT – compiled	F1– P0+F1 exposure	2% glucose		0.7544

Table S3 Hypoxia exposure reduces neutral lipids in the initial exposure, causes an intergenerational decrease in naïve progeny and repeated generational hypoxia exposure eliminates any effect on lipids as assessed by oil red O staining. Last row consists of compiled p-values from the three experiments using Combined Fisher's Test.

Strain + replicate	Generation	Condition	Mean +/- SEM	P values
WT – replicate 1	1st Exposure	Control	167.54 +/- 6.28	
WT – replicate 1	1st Exposure	1% glucose	125.749 +/- 23.86	0.026
WT – replicate 1	1st Exposure	2% glucose	148.25 +/- 8.052	0.2722
WT – replicate 1	2nd Exposure	Control	188.708 +/- 7.17	
WT – replicate 1	2nd Exposure	1% glucose	161.835 +/- 7.162	0.0072
WT – replicate 1	2nd Exposure	2% glucose	173.418 +/- 6.303	0.0954
WT – replicate 1	3rd Exposure	Control	140.75 +/- 33.087	
WT – replicate 1	3rd Exposure	1% glucose	179.75 +/- 3.815	0.0928
WT – replicate 1	3rd Exposure	2% glucose	174.878 +/- 5.761	0.1371
WT – replicate 2	1st Exposure	Control	185.51 +/- 7.968	
WT – replicate 2	1st Exposure	1% glucose	155.773 +/- 7.728	0.035
WT – replicate 2	1st Exposure	2% glucose	140.285 +/- 2.27	0.0031
WT – replicate 2	2nd Exposure	Control	171.24 +/- 4.299	
WT – replicate 2	2nd Exposure	1% glucose	159.263 +/- 8.572	0.1931
WT – replicate 2	2nd Exposure	2% glucose	143.635 +/- 5.248	0.0067
WT – replicate 2	3rd Exposure	Control	143.17 +/- 6.898	
WT – replicate 2	3rd Exposure	1% glucose	148.273 +/- 9.352	0.6085
WT – replicate 2	3rd Exposure	2% glucose	143.503 +/- 5.677	0.9733
WT – replicate 3	1st Exposure	Control	186.083 +/- 2.462	
WT – replicate 3	1st Exposure	1% glucose	164 +/- 11.667	0.1992
WT – replicate 3	1st Exposure	2% glucose	164.5 +/- 15.548	0.209
WT – replicate 3	2nd Exposure	Control	182.667 +/- 5.825	
WT – replicate 3	2nd Exposure	1% glucose	188.5 +/- 14.144	0.8074
WT – replicate 3	2nd Exposure	2% glucose	175.25 +/- 24.885	0.7567
WT – replicate 3	3rd Exposure	Control	186 +/- 8.738	
WT – replicate 3	3rd Exposure	1% glucose	182.167 +/- 13.313	0.8019
WT – replicate 3	3rd Exposure	2% glucose	175.25 +/- 13.431	0.485
WT – replicate 4	1st Exposure	Control	170.325 +/- 5.669	
WT – replicate 4	1st Exposure	1% glucose	156.548 +/- 9.12	0.2401
WT – replicate 4	1st Exposure	2% glucose	127.403 +/- 6.517	0.0017
WT – replicate 4	2nd Exposure	Control	170.76 +/- 6.862	
WT – replicate 4	2nd Exposure	1% glucose	155.288 +/- 8.023	0.1361
WT – replicate 4	2nd Exposure	2% glucose	134.728 +/- 3.968	0.0023
WT – compiled	1st Exposure	Control		
WT – compiled	1st Exposure	1% glucose		0.01002133
WT – compiled	1st Exposure	2% glucose		0.00020794
WT – compiled	2nd Exposure	Control		
WT – compiled	2nd Exposure	1% glucose		0.02466539
WT – compiled	2nd Exposure	2% glucose		0.00059858
WT – compiled	3rd Exposure	Control		
WT – compiled	3rd Exposure	1% glucose		0.40228146
WT – compiled	3rd Exposure	2% glucose		0.48443153

Table S4 Repeated elevated glucose exposure reduces fertility in the first and second generations but the effect of glucose on fertility is eliminated in the 3rd successive generation. Last row consists of compiled p-values from the three experiments using Combined Fisher's Test.

Table S5 Gene list of significantly dysregulated genes after one exposure to hypoxia
